# DNA aneuploidy and breast cancer: a meta-analysis of 141,163 cases

**DOI:** 10.18632/oncotarget.11130

**Published:** 2016-08-09

**Authors:** Jing Xu, Lei Huang, Jun Li

**Affiliations:** ^1^ Department of Medical Oncology, the First Affiliated Hospital of Anhui Medical University, Hefei, China; ^2^ Department of Gastrointestinal Surgery, the First Affiliated Hospital of Anhui Medical University, Hefei, China; ^3^ Department of Tumor Cytology, the First Affiliated Hospital of Anhui Medical University, Hefei, China; ^4^ German Cancer Research Center (DKFZ), Heidelberg, Germany

**Keywords:** breast cancer, diploidy, aneuploidy, estrogen receptor, survival

## Abstract

**Background & Aims:**

DNA ploidy, a DNA flow cytometry parameter, reflects tumor cell cycle. In breast cancer (BC), ploidy status characterizes genotypic stability and potential metastatic capacity. It is suggested that aneuploidy is an independent prognosticator for BC patients and could aid for individualized medicine. There are extensive studies concerning the prognostic significance of DNA aneuploidy, however, its clinical utility remains controversial. Herein we conducted a meta-analysis to determine the correlation between DNA ploidy status and BC characteristics and survival.

**Methods:**

The electronic databases PubMed, EMBASE, and Web of Science were searched for relevant studies. The major investigated parameters were the BC aneuploidy rates in relation to tumor stage, size, lymph node metastasis, grading, estrogen receptor (ER) status, disease-free survival (DFS), and overall survival (OS). Hazard ratios (HRs) and the corresponding 95% confidence intervals (CIs) for DFS and OS were extracted from each study before meta-analyzed. Risk ratios (RRs) were computed using the fixed-effect or random-effects model according to data heterogeneity, and the Mantel-Haenszel or the inverse-variance method was adopted where appropriate to obtain pooled estimates using RevMan 5.3. The Egger's test was conducted with Stata 11.

**Results:**

Pooled analyses of data from 29 studies involving a total of 141,163 cases showed that BC patients with more advanced tumors (stage I *vs.* stages II-IV, RR=0.84; 95% CI, 0.74 to 0.96; *P*=0.01), larger tumors (≤2 cm *vs.* >2 cm: RR=0.82; 95% CI, 0.77 to 0.87; *P*<0.00001), lymph node metastasis (pN0 *vs.* pN1-3: RR=0.85; 95% CI, 0.83 to 0.87, *P*<0.00001), poorer tumor proliferation (G2 *vs.* G1: RR=1.58; 95% CI, 1.40 to 1.79; *P*<0.00001; G3 *vs.* G1: RR=2.17; 95% CI, 1.77 to 2.67; *P*<0.00001; G3 *vs.* G2: RR=1.41; 95% CI, 1.25 to 1.60; *P*<0.00001), and ER^−^ status (ER^−^ *vs.* ER^+^: RR=1.32; 95% CI, 1.22 to 1.43; *P*<0.00001) were significantly more frequently aneuploid. BC patients with diploid tumors had better clinical outcomes than those with aneuploid cancers. The pooled HR estimates were0.73 (*P*<0.0001) for DFS and 0.72 (*P*=0.0001) for OS, respectively.

**Conclusion:**

This meta-analysis implies that DNA aneuploidy is a significant predictor for BC progression and survival, and should be focused on in the therapeutic planning.

## INTRODUCTION

Breast cancer (BC) is one of the most common malignancies and a leading cause of cancer-related mortality among women worldwide [1, 2]. Tumor size, lymph node (LN) metastasis, and hormone receptor status are the most preferred prognosticators applied by oncologists in the management of BC patients. However, as patients with histologically similar tumors at the same disease stage may have different clinical outcomes, it remains difficult to predict patient survival. Personalized treatment regimens require a precise prediction of individual disease outcome. Accordingly, numerous histopathological features and markers have been introduced [3].

DNA cytometric techniques, such as flow cytometry (FCM) and image DNA cytometry, are generally used in tumor cytology [4]. Clinically, fine needle aspiration (FNA) is listed among the routine cytometric analyses for suspicious breast masses. FNA biopsy is a quick technique for pre-operative diagnosis of BC and the fresh tissue samples obtained could be used for further DNA cytometric analyses [5].

**Figure 1:**
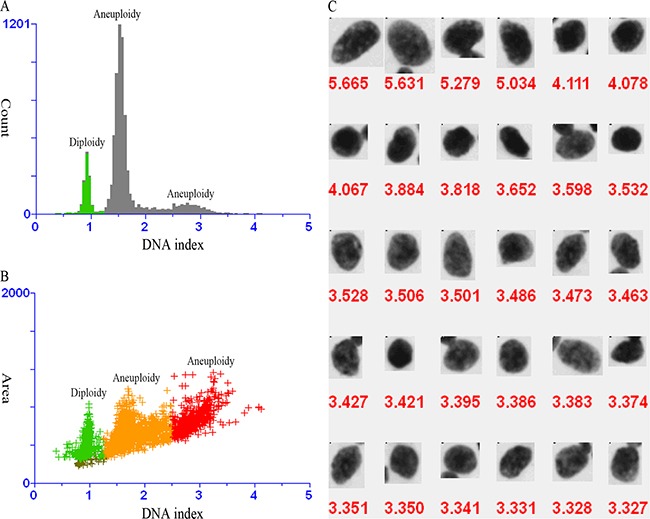
DNA analysis of fine needle aspirates of a suspicious lump **A.** Three separate G0-1 peaks in DNA content histogram (green for diploidy, and grey for aneuploidy). **B.** One diploid peak (green) and two aneuploid peaks (orange and red) in DNA content scatter plot. **C.** Aneuploid cells with DNA index value > 2.5.

As DNA cytometry is widely used in tumor pathology, DNA ploidy emerges as a prominent marker to reflect tumor proliferation. DNA ploidy is the set number of cell chromosomes. Due to the fact that DNA ploidy reflects the cell cycle of a tumor, it is supposed to reflect the biological behavior of the malignancy. DNA ploidy status is mostly defined as follows: the determination of DNA ploidy refers to DNA index (DI), which represents the ratio of the DNA content of G0/1 tumor cells to the reference G0/1 diploid peak (2n) for each specimen as shown in flow cytometry plots (Figure 1). DI = 1 suggests a near diploid specimen, which means only one peak of G0/1 cells in the near diploid region (2n) with few G2M tumor cells in the tetraploid region (4n). On the other hand, if an additional peak with a different DI is present, the tumor is considered aneuploid. Tumors presenting hypo-diploidy (DI < 0.95) and hyper-diploidy (1.04 ≤ DI < 1.44) are classified as aneuploidy BCs [6].

Various studies [7–35] have suggested that DNA ploidy might be associated with recurrence risk and mortality in BC, but their results were mixed, giving controversial views on cancer treatment. Herein we carried out a meta-analysis on the association between DNA ploidy status and BC characteristics and survival.

## RESULTS

### Study selection

**Figure 2:**
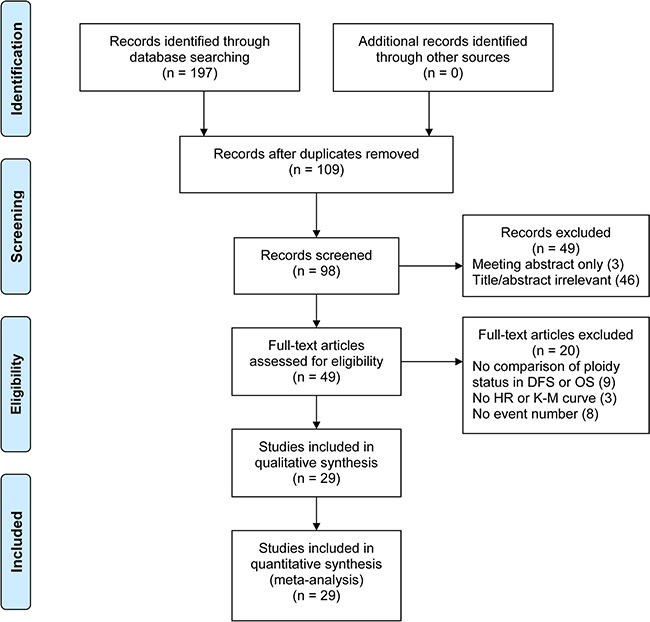
PRISMA flow diagram DFS, disease-free survival; OS, overall survival; HR, hazard ratio; K-M, Kaplan-Meier.

A total of 49 studies were retrieved for full review during primary search according to the inclusion criteria, and 29 studies [7–35] were finally selected (Figure 2). The characteristics of each study were listed in Table 1.

**Table 1:** Characteristics of studies included in this meta-analysis

Authors	Year	No. of patients	Age (year)	TNM stage	Follow-up month^#^	Mastectomy	BCT	HT	CT	HT & CT	NOS
Kallioniemi *et al.*	1987	308	NR	I-III	96^**^	100	53	NR	48	NR	7
Clark *et al.*	1989	395	> 50	N0	59	NR	NR	NR	NR	NR	8
Muss *et al.*	1989	101	59 (27-83)	N0	51	NR	NR	0	0	0	7
Toikkanen *et al.*	1989	351	56 (30-89)	I-IV	324 (264-504)	203	NR	NR	NR	NR	7
Sigurdsson *et al.*	1990	367	62 (21-96)	N0	48 (24-70)	323	44	83	9	10	7
Winchester *et al.*	1990	257	NR	N0	80 (1-148)	NR	NR	NR	NR	NR	8
Beerman *et al.*	1990	690	57 ± 14	I-III	84	NR	NR	NR	NR	NR	7
Keyhani-Rofagha *et al.*	1990	165	58 (27-81)	N0	103; 97^*^	165	NR	NR	NR	NR	7
Merkel *et al.*	1993	326	NR	N0	76; 78 ± 2^*^	NR	NR	0	0	0	7
Wenger *et al.*	1993	127000	NR	NR	26	NR	NR	NR	NR	NR	8
Balslev *et al.*	1994	421	NR	N0	81 (19-160)	404	15	0	0	0	8
Bergers *et al.*	1996	932	NR	T1-3	NR	NR	NR	NR	NR	NR	7
Desserich *et al.*	1997	57	44 (29-55)	N0	73.2 (9.6-209)	33	NR	2	13	NR	7
Bergers *et al.*	1997	1301	60 (27-93)	Operable	84^**^	NR	NR	NR	NR	NR	7
Harbeck *et al.*	1999	125	56 (35-82)	N0	72 (47-108)	83	42	0	0	0	7
Pinto *et al.*	1999	308	58.5 (23-88)	T1-3N0-1	39.6 (3-84)	208	28	127	132	NR	8
Chassevent *et al.*	2001	633	55.3(24-75)	T1-2N0-1M0	69	125	508	163	139	NR	7
Pinto *et al.*	2001	306	58.5 (23-88)	I-II	39.6 (3-84)	278	28	46	84	43	7
Prasad *et al*	2001	332	63 (29-92)	I-IV	120 (84-144)	279	NR	NR	NR	NR	7
Tsutsui *et al.*	2001	653	54.4 (27-93)	N0	46^*^	437	216	219	209	211	7
Pinto *et al.*	2003	392	63 (24-91)	IIB-IV	81 (3-117)	172	3	113	174	NR	8
Tsutsui *et al.*	2003	998	53.1 (25-85)	NR	42	755	243	344	348	306	7
Michels *et al.*	2004	1984	58 (23-93)	NR	54 (1-140)	661	1129	NR	NR	NR	7
Kute *et al.*	2004	556	61.7 ± 13.6	N0	93.6 (1-140.4)	NR	NR	191	161	NR	7
Zabotto *et al.*	2005	271	56 (31-87)	I-II	64 (5-95)	88	183	87	51	85	8
Pinto *et al.*	2006	135	62 (32-83)	T1-2N0	58.5 (6-132)	81	54	87	25	NR	7
Gazic *et al.*	2007	770	60 (22-94)	I-IV	90	475	215	310	314	131	7
Pinto *et al.*	2013	393	59 (23-88)	I-IIIA	134 (50-240)	275	118	121	113	52	8
Pinto *et al.*	2015	684	60 (23-89)	I-IV	134.5 (56-272)	445	239	233	155	158	8

NR, not reported; BCT, breast conserving surgery; HT, hormone therapy; CT, chemotherapy; NOS, Newcastle-Ottawa Scale

# shown as median (range [if given]);

* mean ± standard deviation (if given);

** overall follow-up month.

### Outcomes

The 29 included studies were published between 1987 and 2015, with follow-up periods of 26 to 324 months. A total of 141,163 individuals were included in our analysis. Detailed data and analyses by categories are available in Table 2.

**Table 2:** Analyses of outcomes by categories

Outcome	No. of studies	Participants	Statistical model	Effect estimate (95% CI)
Stage I *vs.* II-IV	6	2315	Random-effects	0.84 (0.74, 0.96)
T1 *vs.* T2-4	11	26570	Random-effects	0.82 (0.77, 0.87)
N0 *vs.* N1-3	8	24763	Fixed-effect	0.85 (0.83, 0.87)
G2 *vs.* G1	8	1804	Fixed-effect	1.58 (1.40, 1.79)
G3 *vs.* G1	8	1542	Random-effects	2.17 (1.77, 2.67)
G3 *vs.* G2	8	2206	Random-effects	1.41 (1.25, 1.60)
ER^−^ *vs.* ER^+^	12	117988	Random-effects	1.32 (1.22, 1.43)
Age < 50 *vs.* ≥ 50 years	8	27209	Random-effects	1.00 (0.91, 1.11)
Age < 40 *vs.* ≥ 40 years	3	1103	Fixed-effect	0.99 (0.81, 1.21)
Pre- *vs.* post-menopausal	4	1990	Random-effects	1.02 (0.93, 1.12)

CI, confidence interval.

#### Aneuploidy rate with regard to tumor stage

**Figure 3:**
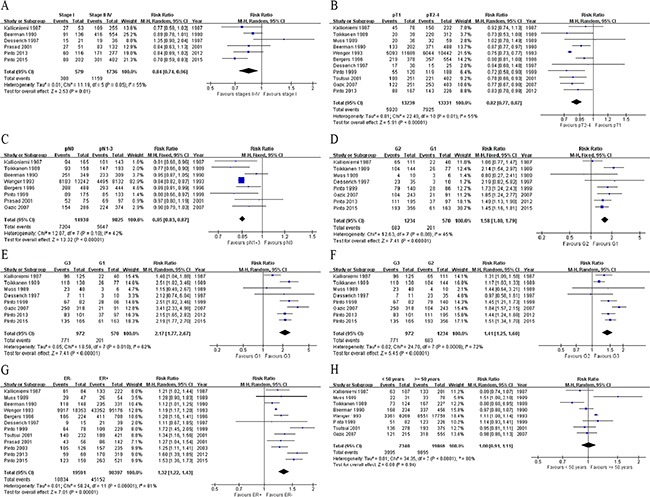
Forest plots of ploidy status vs. BC pathologic features and age Aneuploidy is significantly more frequent in stage I tumors than in stages II-IV ones **A.** in > 2 cm tumors than in ≤ 2 cm ones **B.** in tumors with lymph node metastasis than in pN0 ones **C.** in G2 tumors than in G1 tumors **D.** in G3 tumors than in G1 tumors **E.** in G3 tumors than in G2 tumors **F.** and in ER^−^ tumors than in ER^+^ tumors **G.** However, no significant differences exist between patients ≥ 50 years and those < 50 years **H.** ER, estrogen receptor; M-H, Mantel-Haenszel; CI, confidence interval.

Six studies [7, 11, 20, 25, 34, 35] investigated the correlation between aneuploidy rate and tumor pTNM stages. This pooled analysis revealed that aneuploidy was significantly less frequent in patients with stage I BC compared to those with stages II-IV tumors (RR = 0.84; 95% CI, 0.74 to 0.96; *P* = 0.01, random-effects model; Figure 3A; sign test: *P* = 1; RD = −0.12; 95% CI, −0.17 to −0.08; *P* < 0.00001, fixed- effect model). The result from the Egger's test showed that there was no indication of a bias (*P* = 0.956).

#### Aneuploidy rate with regard to tumor size

Eleven studies [7, 9–11, 16, 18, 20, 22, 26, 33, 34] examined the correlation between aneuploidy rate and tumor size. This meta-analysis revealed that aneuploidy was significantly less frequent in ≤ 2 cm tumors than in > 2 cm ones (RR = 0.82; 95% CI, 0.77 to 0.87; *P* < 0.00001, random-effects model; Figure 3B; sign test: *P =* 0.013; RD = −0.14; 95% CI, −0.15 to −0.13; *P* < 0.00001, fixed-effect model). The Egger's test showed that there was no significant bias (*P* = 0.408).

#### Aneuploidy rate with regard to LN status

There are 8 studies [7, 10, 11, 16, 18, 22, 25, 33] examining the correlation between aneuploidy and tumor pN status. The pooled data implied that aneuploidy was significantly less frequent in BCs with pN0 than in LN metastasis ones (RR = 0.85; 95% CI, 0.83 to 0.87; *P* < 0.00001; sign test: *P =* 0.074; Figure 3C; RD = −0.09; 95% CI, −0.10 to −0.07; *P* < 0.00001), based on a fixed-effect model.

#### Aneuploidy rate with regard to tumor grading

Eight studies [7, 9, 10, 20, 22, 33–35] provided data concerning the association between aneuploidy frequency and tumor grade. The meta-analysis revealed that aneuploidy was significantly more frequent in G2 tumors than in G1 tumors (RR = 1.58; 95% CI, 1.40 to 1.79; *P* < 0.00001, fixed-effect model; Figure 3D; sign test: *P =* 0.074; RD = 0.21; 95% CI, 0.13 to 0.28; *P* < 0.00001, random-effects model). Besides, aneuploidy was significantly more frequent in G3 tumors than in G1 tumors (RR = 2.17; 95% CI, 1.77 to 2.67; *P* < 0.00001, random-effects model; Figure 3E; sign test: *P =* 0.041; RD = 0.44; 95% CI, 0.36 to 0.52; *P* < 0.00001, random-effects model). Similar results were observed when comparing G3 and G2 tumors (RR = 1.41; 95% CI, 1.25 to 1.60; *P* < 0.00001; Figure 3F; sign test: *P =* 0.041; RD = 0.23; 95% CI, 0.16 to 0.30; *P* < 0.00001) based on a random-effects model. The results from the Egger's tests showed that there was no indication of biases for these two meta-analyses (*P* = 0.316 and 0.437, respectively).

#### Aneuploidy rate with regard to ER status

Twelve studies [7, 9, 11, 16, 18, 20, 22, 25–27, 34, 35] investigated the aneuploidy frequency in ER^+^ and ER^−^ BCs. The pooled analysis uncovered that aneuploidy was significantly more frequent in ER^−^ tumors than in ER^+^ tumors (RR = 1.32; 95% CI, 1.22 to 1.43; *P* < 0.00001; Figure 3G; sign test: *P =* 0.04; RD = 0.18; 95% CI, 0.12 to 0.24; *P* < 0.00001) based on a random-effects model. The Egger's test demonstrated no significant bias (*P* = 0.908).

#### Aneuploidy rate with regard to age and menopausal status

Analyses of the association between aneuploidy and age revealed no statistical significance. From eight [7, 9–11, 16, 22, 26, 33] studies using 50 years of age as a cut point, the RR of patients with aneuploidy tumor less than 50 years compared to those above 50 years old was 1.00 (95% CI, 0.91 to 1.11; *P* = 0.94; Figure 3H). Likewise, pooled analysis of the studies [20, 26, 34] using 40 years as a cut point showed a similar result (RR = 0.99; 95% CI, 0.81 to 1.21; *P* = 0.92). Four studies [7, 17, 18, 34] explored the aneuploidy frequency in premenopausal and postmenopausal women with BCs and our analysis demonstrated that aneuploidy was not significantly associated with menopausal status (RR = 1.02; 95% CI, 0.93 to 1.12; *P* = 0.66; sign test: *P =* 0.04). Egger's tests showed that there was no bias for these meta-analyses (*P* = 0.146, 0.365, and 0.365, respectively).

#### DFS and OS of BC in relation to ploidy status

**Figure 4:**
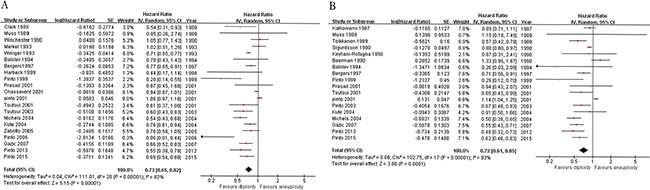
Forest plots of ploidy status vs. survival in breast cancer Patients with aneuploid tumors have significantly worse disease-free survival **A.** and overall survival **B.** compared with diploid ones. IV, inverse variance; CI, confidence interval.

Patients with aneuploid tumors had significantly worse DFS (21 studies [8, 9, 14–17, 19, 21–26, 28–35]; HR = 0.73; 95% CI, 0.65 to 0.82; *P* < 0.00001; Figure 4A; sign test: *P =* 0.001) and OS (18 studies [7, 9–13, 17, 19, 22, 24–27, 29, 30, 33–35]; HR = 0.72; 95% CI, 0.61 to 0.85, *P* =0.0001; Figure 4B; sign test: *P =* 0.016) compared with diploid ones, based on a random-effects model.

### Subgroup analysis

Subgroup analysis was carried out and focused on node-negative BC. Pooled results showed that aneuploidy remained significantly more prevalent in ≤ 2 cm tumors compared to > 2 cm ones (RR = 0.82; 95% CI, 0.71 to 0.95; *P* = 0.009), and in ER^−^ BCs than ER^+^ ones (RR = 1.32; 95% CI, 1.16 to 1.51; *P* < 0.00001) based on a fixed-effect model. The pooled HR for OS was 0.87 (6 studies [9, 12, 13, 17, 26, 29], 95% CI, 0.79 to 0.95, *P =* 0.002; sign test: *P =* 0.480) based on the fixed-effect model. The pooled HR for DFS was 0.76 (9 studies [8, 9, 14, 15, 17, 21, 26, 29, 32], 95% CI, 0.60 to 0.96, *P =* 0.02; sign test: *P =* 0.134) based on the random-effects model. There is no indication of biases for these subgroup analyses (Egger's test: *P* = 0.332, 0.058, and 0.392, respectively).

### Sensitivity test

Sensitivity analyses were performed for all the outcomes. Particularly, the sensitivity test excluding the largest study [16] showed that the effect estimate remained very similar (tumor size: RR = 0.84, 95% CI, 0.79 to 0.88, *P <* 0.00001; LN metastasis: RR = 0.88, 95% CI, 0.84 to 0.93, *P <* 0.00001; ER status: RR = 1.34, 95% CI, 1.23 to 1.46, *P <* 0.00001; age with 50 years as cut-point: RR = 0.98, 95% CI, 0.89 to 1.08, *P =* 0.65; DFS: HR = 0.73, 95% CI, 0.64 to 0.83, *P <* 0.00001). In tumor stage analysis, after excluding Wyss-Desserich *et al.*'s study [20], which was the only one focusing on patients with node-negative tumors, the outcome remained alike; however, when Pinto *et al.* (2013)'s data [34] were omitted, the significance was weakened (*P* = 0.06). In tumor grade analyses, after omitting Gazic *et al.*'s study [33], which used tumor samples from fine needle aspirates (FNAs) compared to the others which used tissues from surgery, the outcomes remained significant (G3 *vs.* G1: RR = 2.12, 95% CI, 1.88 to 2.39; *P* < 0.00001; fixed-effect model; G3 *vs.* G2: RR = 1.37, 95% CI, 1.28 to 1.46; *P* < 0.00001; fixed-effect model). Sensitivity analyses of the other outcomes yielded similar results. Egger's test and an exhaustive literature search conferred a substantial degree of confidence in our pooled findings.

## DISCUSSION

DNA content analysis has been suggested to assess cell kinetics insofar as the DNA percent of S phase cells is identified by histogram. However, the S phase assessment is usually hampered by the overlap between aneuploid tumor and diploid non-malignant cell populations, few cells, background debris, and multiploid/hypertetraploid tumors. For this reason, in human tumor cells, DNA content analysis is mainly used to evaluate the occurrence of aneuploid cell population, an abnormality known to represent the tumor cells. Though the role of aneuploidy as an independent prognostic parameter in BC has been widely studied during the last decades, the results remained controversial. This study is the first meta-analysis focusing on this particular aspect of aneuploidy in BC. It shows that the frequency of DNA aneuploidy is significantly associated with certain prognostic factors, such as tumor size, grade, LN metastasis, and ER status in BC patients. Aneuploidy is more frequently detected in BCs with larger diameters, poorly differentiated cells, LN metastasis, and negative ER expression. However, it was not significantly correlated with menopausal status or age. This study also supports aneuploidy as a significant prognosticator for DFS and OS in BC. Patients with diploid tumors benefited from a significantly reduced risk (27% and 28%, respectively) for cancer recurrence and death.

It is well described that cancer develops from normal tissue through adenoma to carcinoma, and finally metastasis. Aneuploidy has been suggested to be related to cell proliferation and poor differentiation but not disease stage [36]. Our analysis showed a significantly positive correlation between aneuploidy and BC stage, as well as tumor size. It implies the potential role of aneuploidy in BC progression. LN metastasis is a high-risk factor for BC. Previous studies revealed controversial results of the correlation between DNA aneuploidy and LN metastasis. Some of the studies have failed to demonstrate the relationship between ploidy and node status [11, 18, 25, 37]. Based on 8 studies on this aspect, our analysis showed a significant association between aneuploidy and LN metastasis. In addition, aneuploidy was shown to be correlated with a poor survival, especially in node-negative BC. Furthermore, Fallenius *et al.* [38] demonstrated that node-positive non-aneuploid tumors entailed a better survival than node-negative but aneuploid tumors, suggesting that aneuploidy in this cohort was a stronger prognostic marker than node assessment. Thus, aneuploidy might be a BC prognosticator and provide a valuable clue for early intervention. The relationship between DNA ploidy and BC grade has been reported in many studies [6, 39]. The result of this analysis reveals that aneuploidy is more frequent as tumor grade increases. Therefore, it is suggested that patients with well differentiated or diploid tumors tend to have relatively good clinical outcomes. Base on the fact that the extension of disease remains the most significant prognostic factor in BC, aneuploidy may have a prognostic impact within each stage of the disease. For patients with G2 BCs, the therapeutic decision making is more difficult because of the relatively equivocal information. In this regard, DNA ploidy status may provide some useful prognostic information. Szasz *et al.* [40] investigated a CIN4 chromosomal instability gene signature by quantitative polymerase chain reaction analysis, and found that tumor aneuploidy stratifies G2 tumors into good and poor prognosis groups. Pinto *et al.* [32] also carried out a study focusing on this subset of aneuploidy, and found that aneuploidy is negatively correlated with DFS in T1-2N0-2 G2 BC patients. Thus patients diagnosed with aneuploidy BC should be classified into high-risk groups and require systematic treatment. More studies exploring the outcomes of aneuploidy or diploidy on G2 BC are needed.

There is a close correlation between DNA ploidy and ER status. Due to the biological and clinical significances of DNA ploidy, it should be focused on during therapeutic planning. Systemic chemotherapy is recommended for the majority of BCs [41]. However, previous studies [41, 42] had inconsistent results on the benefit of chemotherapy for patients with different ER statuses. Our results show that aneuploidy occurs more frequently in ER^−^ BC, especially node-negative ER^−^ BC, which implies that, adjuvant chemotherapy should be selected for this subgroup of patients. And for those with ER^+^ tumors, appropriate hormone therapy could be applied.

The outcomes of BC patients might be affected by treatment modalities. However, this analysis demonstrates that patients with aneuploid tumors perform worse than those with diploidy ones, even though they are supplied with adjuvant therapy. In 2005, Moureau-Zabotto *et al.* [31] carried out a study on the predictive value of aneuploidy comparing patients receiving adjuvant chemotherapy with those without chemotherapy. In patients who received adjuvant chemotherapy, DNA ploidy status before treatment had no correlations with DFS and OS. On the contrary, in patients without chemotherapy, aneuploid tumors had significantly worse DFS and OS compared to diploid ones. Therefore, aneuploidy might be used as a recurrence prognosticator for therapeutic decision making. Furthermore, BC polyploidy was shown to be correlated with induction of stemness (conversion of usual BC cells into cancer stem cells). Particularly, polyploidy and stemness might be enhanced after treatment with irradiation or chemotherapy [43–46]. The addition of (neo)adjuvant therapy might be harmful to some patients. Thus, DNA flow cytometry might be needed during the therapeutic process to revise treatment strategies. Since more patients are now receiving adjuvant treatment, randomized studies with larger series of participants are needed to further determine the value of DNA ploidy status in anti-BC therapy.

Besides, DNA ploidy analysis using the DNA flow cytometry method has its advantages. Most molecular/genomic technologies are not applied in routine practice [47, 48]. There exist difficulties in data interpretation, significant cost, and lack of standardization [49, 50]. And tumor grading requires the assessment by experienced pathologists. Comparably, DNA ploidy analysis by flow cytometry is a simple, fast, cheap, and standardized method [47, 48, 51]. By following recommended protocols and using appropriate controls, the error's possibility would be minimized and data would become more precise and reproducible.

The strengths of our analyses lie in the thorough literature research and qualified statistical approaches, which provided the veritable results. Besides, all the selected studies are of relatively high quality. However, this meta-analysis has some limitations. First, it is only based on published but not individual patient data. Second, the studies included were not randomized studies. Third, not all outcome measures were reported by all enrolled studies. Hitherto, the results should be taken into consideration with cautiousness. Further studies are required in this field.

In conclusion, this meta-analysis reveals that DNA ploidy status might refine the outcome assessment and personalized treatment choice. And a significantly higher frequency of aneuploidy at advanced tumor stages implies an increased genomic instability during BC progression. This finding may have important therapeutic implications in BC. Furthermore, it should be noted that the value of aneuploidy for BC prognosis needs to be validated in further multicenter studies with larger samples and longer follow-up periods. This would be, undoubtedly, a giant step forward to improve the personalized medicine in BC management.

## METHODS

### Publication search

This meta-analysis was guided by the Preferred Reported Items for Systematic Reviews and Meta-Analysis (PRISMA) statement issued in 2009 [52]. The electronic databases PubMed, EMBASE, and Web of Science were searched for relevant published studies up to November 20^th^ 2015, using the following keywords: ‘DNA ploidy/aneuploidy’, ‘cytometry’, and ‘breast/mammary cancer/carcinoma’. The American Society of Clinical Oncology annual meeting abstract was also retrieved. The language limitation was not applied during search.

### Inclusion criteria

To be considered eligible for this meta-analysis, the relevant clinical studies were carefully selected based on the following criteria: 1) available baseline statuses of enrolled women; 2) estimation of overall survival (OS), disease free survival (DFS), or relapse free survival (RFS)-DNA content relationship as aim of the study; 3) patients were diagnosed BC by histological method and the specimens were collected before any anticancer treatment (*e.g.*, chemotherapy, radiotherapy, hormone therapy); 4) clear definition of DNA diploidy and aneuploidy; 5) the Kaplan-Meier (K-M) method was used to estimate survival rate according to the DNA content, and a Cox proportional hazard regression model was occupied to investigate the relative strength and independent prognostic value of the variables. Alternatively, the study reported hazard ratio (HR) with a 95% confidence interval (CI).

Xu J and Huang L implemented the literature search, and identified eligible papers according to the inclusion criteria. Li J participated in a final decision if consensus could not be reached through discussion. Studies were excluded from the analysis if the retrieved paper was an earlier report of data updated in a subsequent publication.

### Data extraction and definition

Data extraction and quality assessment were conducted by Xu J and Huang L. The data extracted from each eligible study included authors' names, publication year, baseline characteristics, statistic method, DNA ploidy status, median follow-up, adjuvant settings, and survival. The investigated parameters were the BC aneuploidy rates in relation to tumor size, grade, LN metastasis, estrogen receptor (ER) status, DFS, and OS. DFS events included local and distant recurrences of the original cancer, second primary BC, and death. Hazard ratios (HRs) with 95% confidence intervals (CIs) were applied where possible. If HRs and 95% CIs were not directly reported, we extracted data from K-M curves by Engauge Digitizer 4.1 and then calculated the indexes.

### Statistical analysis

Statistical analyses were carried out following the recommendations of the Cochrane Collaboration Guidelines. The quality of included studies was assessed by the Newcastle-Ottawa Scale (NOS) scale. Fixed-effect or random-effects model chosen according to data heterogeneity was adopted to calculate the pooled estimate (risk ratio [RR], risk difference [RD], or HR) using the Mantel-Haenszel's or the inverse variance method based on the available data type. Sign tests were performed to assess the significance of evidence in each dataset. Cochran's Q statistic was used to check the homogeneity assumption. We calculated the Higgins' *I*^2^ index, which describes the percentage of total cross-study variation due to heterogeneity rather than chance, to obtain a quantitative measure of the degree of inconsistency in the results of studies. The fixed-effect model was adopted if no heterogeneity was present (*χ^2^*P** < 0.100 and *I^2^* < 50%). When there existed excessive heterogeneity, data were first rechecked for validation. If the heterogeneity persisted, the DerSimonian random-effects model was employed [53, 54]. The forest plot was generated to display results, and potential publication bias was estimated by Egger's test [55]. Sensitivity test was carried out to investigate the influence of a single study on the pooled estimates by sequentially excluding each study. Subgroup analyses were performed by pooling estimates for similar subsets of patients across studies where available. All statistical analyses were performed using RevMan 5.3 and Stata 11. All *P* values were two-sided.
